# Differential Neutrophil and Eosinophil Infiltrations in the Sub-Lining Compartment of Rheumatoid Versus Osteoarthritic Synovium

**DOI:** 10.31138/mjr.050225.hre

**Published:** 2025-12-31

**Authors:** Maria Sakkou, Anastasios Mourikis, Ilias Fanourgiakis, Alkiviadis Vossos, Maria Tektonidou, George E. Fragoulis, Petros P. Sfikakis

**Affiliations:** 1 Centre of New Biotechnologies & Precision Medicine, School of Medicine, National and Kapodistrian University of Athens, Athens, Greece;; 2 Third Orthopaedic Dept. KAT Attica General Hospital, Greece;; 3 Joint Rheumatology Program, National and Kapodistrian University of Athens Medical School, Athens, Greece

**Keywords:** rheumatoid arthritis, osteoarthritis, synovium, neutrophils, eosinophils, fibroblasts

## Abstract

**Background:**

The role of innate immunity in the perpetuation of synovial inflammation may have been overlooked in rheumatoid arthritis (RA). Herein, we compared and quantified neutrophil and eosinophil infiltrations in lining and sub-lining compartments of RA versus osteoarthritis (OA) synovium.

**Methods:**

Synovia were obtained from consecutive RA and OA patients (n=8, each) with destructive knee arthritis who underwent arthroplasty. Histological stainings and fluorescence immune-stainings were performed on paraffin tissue sections. Neutrophils and eosinophils were imaged with a confocal microscopy using the pan-leukocyte marker anti-CD45 combined with anti-CD14 and anti-CD294 specific antibodies, respectively. Antibodies against CD90 were used for neutrophil-fibroblast and/or eosinophil- sub-lining fibroblast tissue co-localisation analysis.

**Results:**

In both RA and OA patients, higher numbers of leukocytes were quantified in the sub-lining vs lining synovial compartment. In RA, the numbers of neutrophils were higher in the sub-lining vs lining compartment, being increased by 5-fold, which was not the case in OA. On the other hand, eosinophils could be exclusively found in the sub-lining compartment of either RA or OA-derived synovium, being increased in RA. Concerning their possible function we found that both neutrophils and eosinophils could be found in proximity with sub-lining fibroblasts in RA-derived synovia, but not in OA, indicating a crosstalk between these leukocytes and RA synovial fibroblasts.

**Conclusion:**

Increased neutrophilic infiltrations and the presence of eosinophils in synovial sub-lining compartment discriminate RA from OA. Studies to elucidate the contribution of innate immunity cells to the pathophysiology of joint destruction in RA through interactions with fibroblasts are warranted.

## INTRODUCTION

Rheumatoid Arthritis (RA) is a systemic autoimmune disease characterised by cellular and ECM qualitative chronic changes specifically in synovial tissue.^1^ In RA increased infiltrating lymphocytes encounter hyper-proliferating resident lymphocytes and fibroblasts in the synovial membrane leading to chronic, non-resolving localised inflammation in the joints.^2,3^ Furthermore, qualitative ECM changes in the synovial membrane lead to tissue destruction in the affected joints. Latest single cell analysis studies have shown that both immune and fibroblasts in the inflamed joints undergo qualitative changes, adopt an “activated” pathogenic gene expression repertoire and suppress the resolution of local inflammation.^4,5^

The synovium is an intricate tissue anatomically distinguished into a lining compartment, a one to two cell thick layer of cells, situated in close proximity to the articular cartilage and bone and the sublining compartment, a loose thicker cell layer.^1,6^ The synovium in homeostatic conditions is comprised by various cell subpopulations, including fibroblasts, resident macrophages, endothelial cells neurons. However, in RA the synovium increases massively in size and acquires pathogenic pro-inflammatory and pro-destructive features. Deep phenotyping of the RA synovial tissue led to the identification of three RA pathotypes.^1,6,7^ the lympho-myeloid, characterised by the predominant infiltration of B/T cells forming ectopic lymphoid structures in the sub-lining; the diffuse-myeloid, identified by a predominant infiltration of CD68^+^ monocytic cells without distinctly organised follicular structures; and pauci-immune, defined by immune cell infiltrates and synovial fibroblast hyper-proliferation.^8–10^

Over the last years, it has been recognised that within the hypertrophic-aggressive pannus formed in the RA joints, both fibroblasts that release tissue degrading enzymes and macrophages orchestrate disease progression and chronicity. However, latest studies have also uncovered the unique role of innate immune cells like neutrophils and eosinophiles play in the articular microenvironment during RA pathogenesis.^11,12^ Previous studies have shown that in the RA synovium, effector cell states of B and T leukocytes are the main contributors of the chronic- non-resolving synovial inflammation I RA. However, single-cell analysis studies of circulating leukocytes have revealed distinct roles of pathogenic RA neutrophil and eosinophil cell states, especially in patients with seronegative RA.^13–15^ In fact, it has been suggested that neutrophils interact with fibroblastic cell states in the synovium,^15^ and thereby orchestrate further immune responses by producing inflammatory cytokines and chemokines.^15,16^ Moreover, neutrophils have been demonstrated to form neutrophil extracellular traps (NETs), an additional is mechanism possibly contributing to the chronicity of articular inflammation in the RA joint.^7^ Eosinophils are also thought to play a role in the pathogenesis of RA with preliminary findings suggesting that they may play a protective role in active arthritis.^17^

Genetic and clinical phenotype in disease activity and duration vary between RA patients. However, it is evident that if left untreated, it can lead to significant morbidity and devastating consequences including destruction of the joints and disability.^18,19^ This aetiology of disability becomes more complex in some individuals, because of the inevitable co-existent of degenerative osteoarthritis (OA).^20^ The latter often is included in the differential diagnosis of destructive RA, but the underlying pathogenetic mechanisms are largely different, with RA being a classic autoimmune disease and OA being mostly a degenerative disorder.^21^ Yet, un-resolved chronic inflammation (in different degrees) as a net effect in the synovium is also present in OA, as evidenced not only by histopathological studies but also of the beneficial effects of anti-inflammatory agents.^22^ Herein, to gain further insights into the histopathology of destructive arthritis we aimed to compare the histo-pathology between RA and OA RA synovium, focusing on the neutrophil and eosinophile infiltrations.

## METHODS

### Patients’ recruitment and tissue processing

Consecutive patients with a diagnosis of RA (n = 8) and OA (n = 8) undergoing knee arthroplasty as per clinical practice were enrolled in the study. Synovial tissue was detached from the whole joint and removed during arthroplasty. Clinical data, including age, sex, site of the procedure, and complete blood counts were collected for each patient.

To avoid potential bias related to different joint sizes, and origin the analysis involving comparison in OA and RA was performed on patients whose synovial tissue was retrieved from the knee joint only. Synovial tissue was subsequently isolated by removal of any fat tissue attached and section and cut in 100-200 mg of weight fragments. Two randomly picked fragments were fixed O/N in 4% PFA and embedded in paraffin for histological characterisation. One block per patient was prepared.^23^ All patients gave written informed consent and the study was approved by the local Ethics and Scientific Committees of the University Hospital “Laiko” of the National and Kapodistrian University of Athens (No.314/2021).

### Histology and immunohistochemistry

Sequential 5-µm-thick sections of synovial tissue underwent haematoxylin and eosin (H&E) staining to assess the quality of the tissue, determine the level of inflammation, and the degree of cellular infiltration by leukocytes. H&E-stained sections were digitally scanned using Zeiss Axioscan 7.^24,25^

For immune fluorescence processed slides, prior to staining, FFPE sections were rehydrated, and antigen retrieved in 10 mM Sodium Citrate buffer (pH 6.0) for 20 min at 96°C and further 20 min at RT. Slides were subsequently rehydrated with PBS before blocking with 10% normal donkey serum. Primary antibodies were then applied in PBS-10% BSA, THY1/CD90 (sheep polyclonal, AF2067, Bio-Techne), CD45 (clone D3F8Q, Cell Signaling), PDPN (NZ-1.3, eBioscience, PE), CD294 (PA5-20333, Thermo Fisher Scientific) and LY6G (ab122501, Abcam). Appropriate isotype controls were used on separate sequential sections. Secondary antibodies included Donkey anti-Rabbit IgG (H+L) Highly Cross-Adsorbed Secondary Antibody, Alexa Fluor™ 488 (Invitrogen- A-21206), Donkey anti-Sheep IgG (H+L) Cross-Adsorbed Secondary Antibody, Alexa Fluor™ 647 (Invitrogen, A-21448), Donkey anti-Mouse IgG (H+L) Highly Cross-Adsorbed Secondary Antibody, Alexa Fluor™ 647 (Invitrogen, A-31571), Goat anti-Mouse IgG (H+L) Highly Cross-Adsorbed Secondary Antibody, Alexa Fluor™ 594 (Invitrogen, A-11032). Primary antibodies were titrated and used at a concentration that ranged from 1:100 to 1:1000, depending on the antibody, whereas secondary antibodies were all used at 1:400 dilution. Slides were mounted in prolong diamond (Thermo Fisher) and stored at 4°C before imaging. Images were obtained using a Leica TCS SP8X White Light Laser confocal system in linear deconvolution or sequential mode and were analysed using ImageJ (FIJI, publicly available NCBI software).^26–28^ The region of interest used for quantitative analysis was the whole field of view imaged with the same objective and both optical and digital zoom (scale bar is indicated in one of the panels, quantified images were processes with the exact same threshold filters prior to quantitation).

### Statistical analysis

Differences were evaluated by the parametric Student’s t test (two groups) or Kruskal Wallis with Dunn’s post-test (multiple groups). Kolmogorov-Smirnov test was used for checking normal distributions. Statistical analyses were performed using GraphPad Prism-v9 software (Graphpad, San Diego, CA, USA). Data are shown as median and p-values <0.05 were considered significant.

## RESULTS

**Figure 1.**
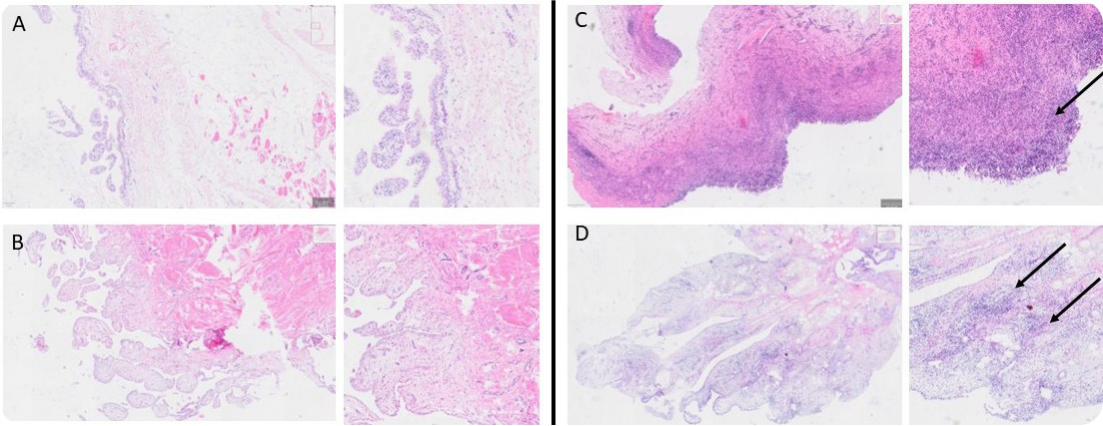
**(above).** Histological Haematoxylin-eosin staining of osteoarthritic (OA) and rheumatoid arthritic synovial membrane (RA). (**A–B**) Representative images of synovial membrane from OA patients, showing low grade infiltration of leukocytes. (**C–D**) In patients with RA, synovium contains strongly visible cell infiltration and lymphoid nodules in some cases (arrow). Magnification ×20.

**Supplementary Figure 1.**
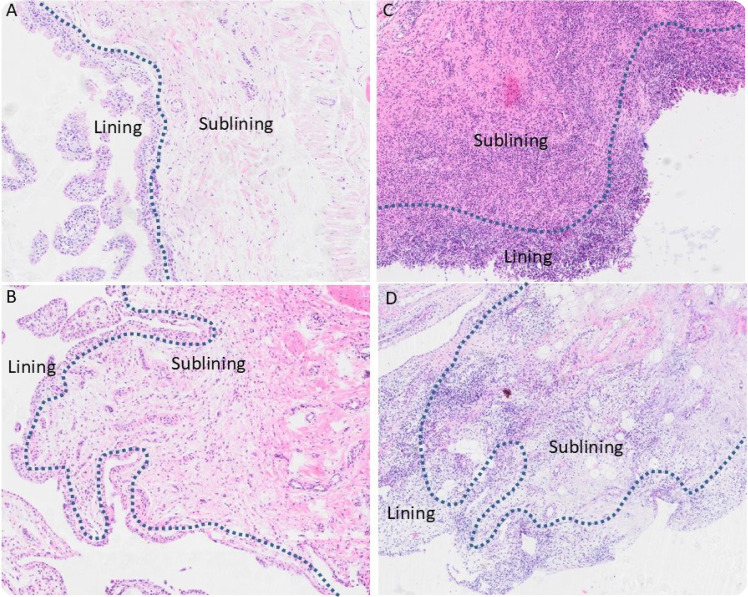
Histological Haematoxylin-eosin staining of osteoarthritic (OA) and rheumatoid arthritis synovial membrane. (RA). Representative higher magnifications of the H&E stainings of the sections from the patients in **Figure 1**.

First, we compared RA and OA synovial tissues collected during knee joint replacement. Characteristics of the patients are depicted in **Supplementary Table-1**. Following H&E staining of synovium represented in **Figure 1A–D** in keeping with previously published data, we observed that the synovial tissue exhibited higher sub-lining and lining cellularity, and higher leukocyte infiltration in RA-derived (**Figure 1C–D, Supplementary Figure 1**) compared to the OA-derived sections (**Figure 1A–B**), which displayed either no synovitis or low-grade synovitis.^29^ Overall, 5 out of 8 OA patients belonged to the low-grade or “no synovitis” group as compared to only 2 out of 8 RA patients respectively.

**Figure 2.**
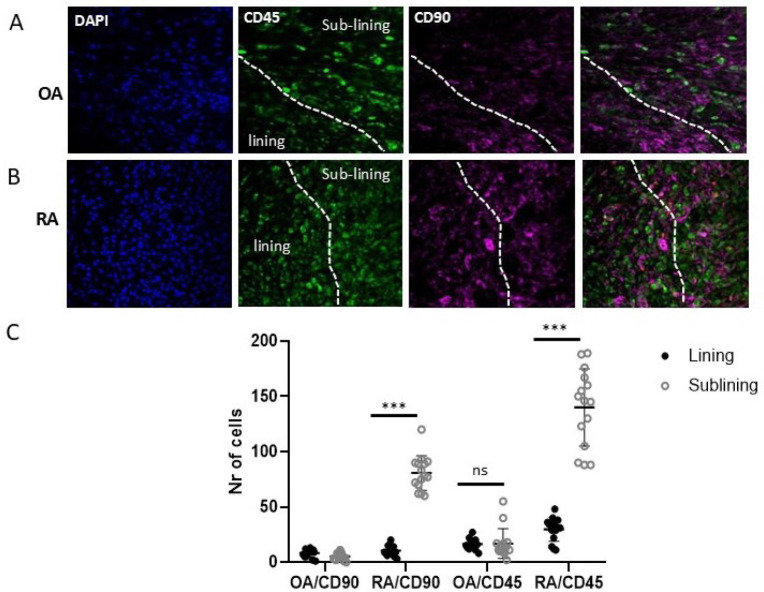
**(left).** Leucocyte synovial infiltration. Representative confocal images from one OA (**A**) and one RA (**B**) patient. DAPI staining indicates the nuclei, leukocytes infiltrates in green (CD45) and resident fibroblasts in magenta (CD90). Lining and sub-lining distinction was performed in the H&E image. Quantitation of the CD90+ fibroblasts and the CD45+ cells was performed on ImageJ and statistical analysis was calculated with Graphpad using Grouped analysis, multiple t-tests significance indicated (**C**), values represent measurements from specified regions-of-interest (ROIs), group t-tests were applied and P value calculated (P<0.000001).

Next, we investigated the presence of leukocyte infiltrates in RA in comparison to OA patients. To examine possible differences in leukocyte infiltration levels we performed confocal microscopy imaging on stained synovium slides with the pan-leukocyte CD45 marker (**Figure 2A–B**). Tissue segmentation on the H&E-stained slides and CD90 staining for sub-lining synovial fibroblasts enabled us to distinguish lining and sub-lining areas on the imaged ranged of interest (ROI) (26,30). We found that more leukocytes, as well as CD90+ fibroblasts, could be identified in the sub-lining compared to lining synovial compartment in the RA patients (mean of 81 cells, vs 10 cells respectively, p<0.0001). Of note, both cell types were found to be increased in the sub-lining compartment in RA (mean of 146 cells) compared to OA (mean of 16 cells), respectively (p<0.0001) (**Figure 2C**).^29^ On the other hand, in the lining compartments CD45+ cell numbers were comparable between RA and OA (mean of 29 versus 16, respectively, p=0.928) (**Figure 2C**).

**Figure 3.**
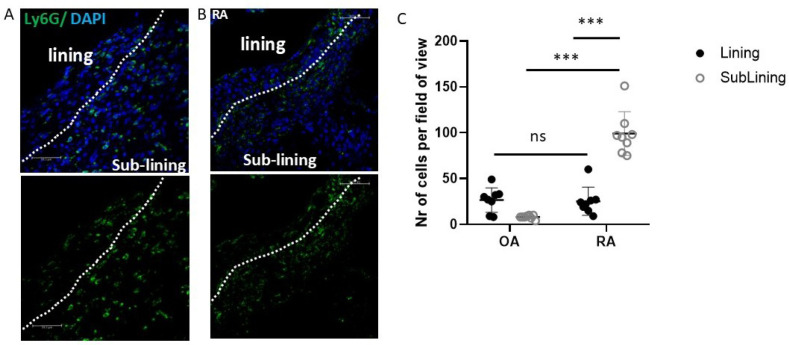
Neutrophil synovial infiltration. Representative confocal images from one OA (**A**) and one RA (B) patient. DAPI staining indicates the nuclei, leukocytes infiltrates in red (Ly6G). Lining and sub-lining distinction was performed in the H&E image. Quantitation of the Ly6G+ cells was performed on ImageJ and statistical analysis was calculated with Graphpad using group t-tests.

To investigate the contribution of neutrophils to the difference in leukocyte infiltration between RA-derived and OA-derived synovia, we quantified CD14+ neutrophils. Ly6G staining^31^ of the synovial sections and confocal imaging analysis revealed that neutro-phil numbers positively associate with that of overall CD45 (**Figure 3A–B**). In RA (but not in OA), the numbers of neutrophils were markedly higher in the sub-lining compared to lining, being markedly increased by 5-fold (in RA mean of 143 cells in the sub-lining and 29 cells in the lining p<0.0001). Compared to RA, samples from OA displayed significantly lower leukocyte infiltration in the sub-lining, (75 cells versus 13 cells on average, respectively) (P= 0.0038) (**Figure 3C**), but not in the lining compartment (OA and RA: mean of 8 and 9 cells/ROI, respectively).

**Figure 4.**
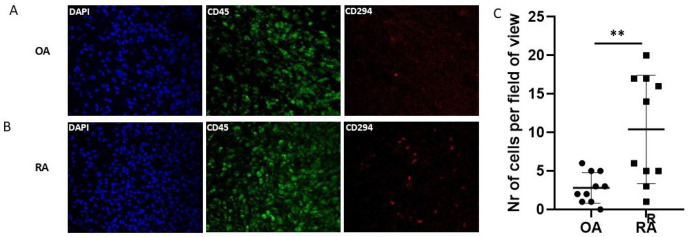
Eosinophile synovial infiltration. Representative confocal images from one OA (**A**) and one RA (**B**) patient. DAPI staining indicates the nuclei, leukocytes infiltrates in green (CD45) and eosinophiles in red (CD294). Lining and sub-lining distinction was performed in the H&E image. Quantitation of the CD294+ cells was performed on ImageJ and statistical analysis was calculated with Graphpad with Students t test (P<0.00001).

We next wanted to examine the presence of eosinophils in RA and OA synovial membranes. Therefore, we identified eosinophils in the tissue section by combining CD45 and the eosinophile- specific gene marker CD294 (**Figure 4A–B**).^32^ In contrast to neutrophils, eosinophils could be exclusively found in the sub-lining compartments, being increased in RA-derived compared to the OA-synovium. Indeed, OA sections displayed very few eosinophiles, i.e. maximum 5 cells have been detected in each ROI quantified (minimum 2 cells, maximum 6), whereas RA sections exerted significantly higher numbers, with an average of 10 cells in each measured ROI (p= 0.0041) (**Figure 4C**).

**Figure 5.**
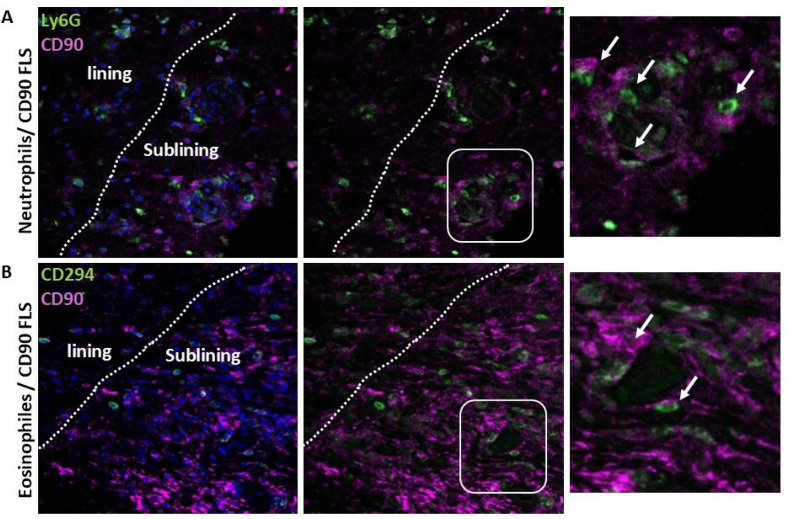
Co-localisation/close proximity of neutrophils and eosinophils with fibroblasts in the sub-lining of the synovium. Representative confocal images immune-stained with CD90 and Ly6G antibodies for co-localisation analysis of neutrophils with fibroblasts (**A**) and CD90 combined with CD294 antibodies for co-localisation analysis of eosinophils to fibroblasts (**B**) in RA inflamed synovium. Insert panels are zoomed in areas of the synovium where co-localisation is detected, as indicated.

**Supplementary Figure 2.**
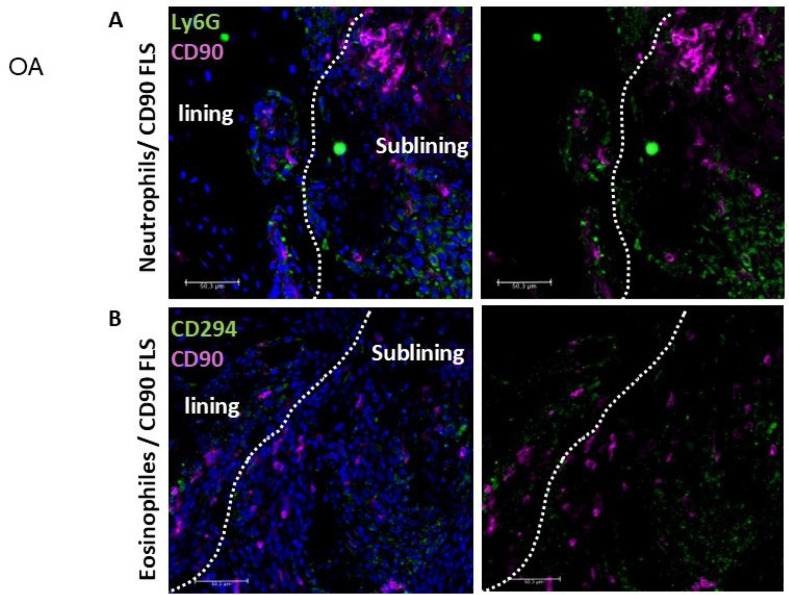
Co-localisation/proximity of neutrophils and eosinophils with fibroblasts in the sub-lining of the synovium in OA patients. Representative confocal images immune-stained with CD90 and Ly6G antibodies for co-localisation analysis of neutrophils with fibroblasts (**A**) and CD90 combined with CD294 antibodies for co-localisation analysis of eosinophils to fibroblasts (**B**) in OA synovium.

Finally, as shown in **Figure 5A–B** (indicated with white arrows), both neutrophils and eosinophils could be found in close proximity with sub-lining fibroblasts in RA-derived, but not in OA-derived synovia (**Supplementary Figure 2A–B**), connoting a possibly functional local crosstalk between these leukocytes and synovial fibroblasts.

## DISCUSSION

Herein, we studied surgical knee specimens from consecutive OA and RA patients with destructive arthritis by confocal microscopy. We performed for the first time a systematic quantitation of neutrophils and eosinophiles in RA and OA affected knee joints. OA is used as control for RA joints in most of the published studies, however in most cases OA joints are also inflammatory tissues, we therefore went on to characterise and quantify the innate immune microenvironment that we believe plays a primary role in the chronicity feature of RA. We show that in RA patients, leukocytic infiltrates were significantly more intense in the sublining compartment of the synovium, compared to lining layer. We also demonstrate that number of neutrophils in the sub-lining compartment of RA specimens was significantly higher to that observed in OA sub-lining. In contrast, numbers of infiltrating eosinophils, were exclusively detected in the sub-lining compartment and were again significantly higher in RA versus OA joints. In RA pathophysiology, the role of neutrophils has been increasingly recognised over the last years. It is well established that RA patients present enhanced NET formation in their extracellular environment.^6,33^ NETs mainly comprise histones, chromatin DNA, and granular proteins, including mainly catabolic enzymes. Neutrophils in the peripheral blood and synovial fluid of RA patients display a propensity of spontaneous formation of NETs. Furthermore, NET formation in RA joint microenvironment is further enhanced by LPS stimulation, in contrast, neutrophils isolated from the blood of OA patients do not.^21^ This implies either that neutrophils in RA patients have been primed to form NETs *in vivo,* which is evident by the presence of NET forming neutrophils in RA synovial tissue, rheumatoid nodules and neutrophilic dermatoses, or that different neutrophil cell states are being recruited in RA compared to OA synovium. On the other hand, the activation of the proinflammatory sub-lining fibroblasts, considered as the key mechanism underlying RA cartilage damage, is multifactorial.^26,29,34^ Here, by demonstrating the proximity of neutrophils to synovial fibroblasts exclusively in the RA we uncovered a possible unique neutrophil-fibroblast interaction that could induce non-resolving inflammation vs OA inflammation. Also, RA synovium has been reported to internalise NET components via the RAGE-TLR9 pathway. This process subsequently induces pro-inflammatory traits in RA synovial fibroblasts, including presentation of the modified (citrullinated) peptides to antigen-specific T cells and thus promote the expansion of pathogenic adaptive immunity, sustaining local joint inflammation and potentially triggering systemic complications.^6,35^ RA joints contain stimuli that are capable of inducing NET formation. Moreover, neutrophils display cytotoxic and proinflammatory properties and it is believed that they contribute as mediators for inflammation and tissue destruction in the synovium.^33,36^

On the other hand, eosinophils display a more elusive role in RA. Eosinophilia is traditionally associated with immune defence mechanisms against hosts, however it has been also implicated in various rheumatologic conditions.^11,17,37^ The prevalence of eosinophilia in RA patients ranges from 3.2 to 21.6% depending on the population and the diagnosis criteria. In some RA eosinophilia is a secondary. Despite the low frequence in RA patients cohorts, some patients also display an increase in circulating levels of eosinophil cationic protein, an eosinophil specific granule protein.^11^ Although it has been suggested that eosinophilia or quantitation of circulating eosinophile derived proteins may be a bad prognostic factor for patients with early RA,^11^ this remains a field of controversy.^12^

The role of eosinophils in the synovial membrane remains obscure. Based on data from RA animal models, it has been hypothesised that inflamed synovia include 2 subtypes of eosinophiles, the classical pro-inflammatory type and a second regulatory subpopulation that plays a pro resolving protective role^33,37^ mitigating inflammation and protecting from bone loss. Towards the same direction, a recent study has found that eosinophils mediate resolution of arthritis in an experimental model and that a specific subset -regulatory eosinophils- were present in the joint of patients with quiescent disease but not in those with disease activity.^17^

Our data establish for the first time the exclusive existence of eosinophiles in RA synovium and a possible interaction between neutrophils and fibroblasts. Regarding the crosstalk between neutrophils or eosinophils and fibroblasts in RA synovium, NETs in the inflamed RA joint have been demonstrated as a source of citrullinated autoantigens leading to RA pathogenic activation and joint damage. At the molecular level, NETs containing citrullinated peptides are internalised by Synovial Fibroblasts through a TLR signalling pathway, thus promoting their pro-inflammatory phenotype and subsequent induction of MHC- II on their cell membrane. Once internalised, arthritogenic NET peptides are loaded into FLS MHC class II. The existence MHCII expressing Synovial Fibroblasts subpopulation has been confirmed by numerous Single-Cell Analysis studies and is positively correlated with increased cartilage damage and worst prognosis in both RA patients and RA genetic models.^35,38^ In contrast, eosinophil function in the joint is poorly studied. In animal models activated eosinophiles have been reported to suppress osteoclasts differentiation by directly regulating their transcriptional profile. These limited data could support a regulatory function of eosinophiles in bone,^17^ however, functional data are not conclusive in RA.

We acknowledge that our study has certain limitations. The number of patients included is relatively low and therefore generalisability of the results is limited. Still, synovial samples -especially via open surgery- are not easily obtained. The low number of patients examined precluded the analysis of possible correlations between the number of infiltrating neutrophils or eosinophils and those circulating in the peripheral blood.

Second, evidence for cell-to-cell interaction is only based on microscopy/imaging findings and third, most of our patients were not naïve, newly diagnosed, and therefore cell-composition in the lesions might have changed. Third, most of our patients (6 out of 8) displayed low disease activity or were on clinical remission and therefore, no correlations could be examined between the number of infiltrating cells in the tissue and the disease activity state or blood inflammation markers (i.e., ESR/CRP).

To conclude, increased neutrophilic infiltrations and the presence of eosinophils in synovial sub-lining compartment discriminate RA from OA. Studies to elucidate the possible contribution of innate immunity cells to the pathophysiology of joint destruction in RA through cell-cell interactions with fibroblasts are warranted.

## Figures and Tables

**Supplementary Table 1.** Characteristics of the patients’ samples used in this study.

	**Rheumatoid arthritis patients (n=8)**	**Osteoarthritis patients (n=8)**
**Features**		
Female Gender, n (%)	6 (75)	6 (75)
Age, (years, mean ± SD)	61.0 ± 10.6	69.8 ± 5.6
Disease Duration, (mean ± SD)	11.1 ± 7.8	NA
Seropositive, n (%)	7 (87.5)	NA
Treatment-naïve, n (%)	3 (37.5)	NA
b/tsDMARDs-naive, n (%)	5 (62.5)	NA

## Data Availability

The data underlying this article will be shared on reasonable request to the corresponding author.
